# Near Infrared Spectroscopy for High-Temporal Resolution Cerebral Physiome Characterization in TBI: A Narrative Review of Techniques, Applications, and Future Directions

**DOI:** 10.3389/fphar.2021.719501

**Published:** 2021-11-05

**Authors:** Alwyn Gomez, Amanjyot Singh Sainbhi, Logan Froese, Carleen Batson, Arsalan Alizadeh, Asher A. Mendelson, Frederick A. Zeiler

**Affiliations:** ^1^ Section of Neurosurgery, Department of Surgery, Rady Faculty of Health Sciences, University of Manitoba, Winnipeg, MB, Canada; ^2^ Department of Human Anatomy and Cell Science, Rady Faculty of Health Sciences, University of Manitoba, Winnipeg, MB, Canada; ^3^ Biomedical Engineering, Faculty of Engineering, University of Manitoba, Winnipeg, MB, Canada; ^4^ Section of Critical Care, Department of Medicine, Rady Faculty of Health Sciences, University of Manitoba, Winnipeg, MB, Canada; ^5^ Centre on Aging, University of Manitoba, Winnipeg, MB, Canada; ^6^ Division of Anaesthesia, Department of Medicine, Addenbrooke’s Hospital, University of Cambridge, Cambridge, United Kingdom

**Keywords:** traumatic brain injury, near-infrared spectroscopy, cerebrovascular reactivity, multi-modal monitoring, precision medicine

## Abstract

Multimodal monitoring has been gaining traction in the critical care of patients following traumatic brain injury (TBI). Through providing a deeper understanding of the individual patient’s comprehensive physiologic state, or “physiome,” following injury, these methods hold the promise of improving personalized care and advancing precision medicine. One of the modalities being explored in TBI care is near-infrared spectroscopy (NIRS), given it’s non-invasive nature and ability to interrogate microvascular and tissue oxygen metabolism. In this narrative review, we begin by discussing the principles of NIRS technology, including spatially, frequency, and time-resolved variants. Subsequently, the applications of NIRS in various phases of clinical care following TBI are explored. These applications include the pre-hospital, intraoperative, neurocritical care, and outpatient/rehabilitation setting. The utility of NIRS to predict functional outcomes and evaluate dysfunctional cerebrovascular reactivity is also discussed. Finally, future applications and potential advancements in NIRS-based physiologic monitoring of TBI patients are presented, with a description of the potential integration with other omics biomarkers.

## Introduction

Traumatic brain injury (TBI) is one of the leading causes of death and disability worldwide, with an annual incidence of approximately sixty-nine million globally (Maas et al., 2008). Despite the evolution of guideline-based management, centered around intracranial pressure (ICP) and cerebral perfusion pressure (CPP) monitoring, little improvement in outcomes have been seen in the last 25 years (Carney et al., 2017; Donnelly et al., 2019). As a result, there has been a growing interest in various multimodal monitoring techniques (Le Roux et al., 2014; Zeiler et al., 2020). These alternative modalities may provide greater insight into the individual patient’s comprehensive physiologic state, or “physiome,” following TBI, resulting in more targeted therapeutic strategies and precision medicine.

One of the emerging technologies in the multimodal monitoring of TBI patients, and the topic of this narrative review, is near infrared spectroscopy (NIRS). The use of NIRS in the monitoring of TBI patients is expanding rapidly, with a recent systematic review finding it to be the most common non-invasive modality alongside transcranial Doppler ultrasonography (TCD; Roldan et al., 2020). This increased clinical adoption is likely in part due to its ease of use, non-invasive nature, and ability to provide continuous monitoring. However, NIRS technology covers a spectrum of different techniques that can be applied to monitor TBI patients. These varying techniques can lead to confusion as the continuous biomarkers of the cerebral physiome provided by each variation are slightly different. As such, we aim to provide a general overview of NIRS and its potential applications in monitoring TBI patients.

This article begins with a review of the fundamental scientific principles of NIRS and presents some of the variations on the technology. Furthermore, it explores the clinical utility of NIRS monitoring through the phases of clinical care of TBI patients, from pre-hospital/emergency department management through to rehabilitation and outpatient follow-up. Its use in the prediction of functional outcomes is also presented. We conclude by examining some of the limitations of the technology, as it stands today, and exploring avenues for future development.

## Principles of Near-Infrared Spectroscopy Technology

### Scientific Basis

The foundational principles of NIRS are based on near infrared (NIR) light, a region in the infrared (IR) spectrum, defined as light having wavelengths ranging from 650 to 950 nm. In 1800, Sir William Herschel first described IR radiation during his experiment of passing sunlight through different filters. This experiment led him to measure the amount of heat generated by each colour in the visible spectrum, where he discovered the increased amount of heat generation beyond the red light. He concluded the existence of invisible rays beyond the visible red light that generate more heat (Herschel, 1800b; Herschel, 1800a). The part of NIR region containing wavelengths between 650 and 950 nm is where the light absorption by water molecules and hemoglobin is low. Otherwise, the visible light is intensely absorbed by hemoglobin below that range, and the absorption of water molecules increases significantly above it. It was not until 1977 that monitoring of cerebral tissue oxygenation and perfusion was described using IR light in NIR range by Franz Jöbsis (Jöbsis, 1977).

To better understand how the NIR light is used to observe blood and tissue oxygenation, we need to look at light absorption. The Beer-Lambert Law describes the absorption of light through a solution. It states that the absorbance of a solution is proportional to its concentration and length of the light path. It is given by Eq. 1.

$$A=\text{log}\left(\frac{I}{I_{0}}\right)=\varepsilon \cdot C\cdot l$$ (1)

In Eq. 1, A is the absorbance of the solution given by logarithmic ratio of transmitted light intensity, I , over incident light intensity, $I_{0}$ . ε is the molar extinction coefficient which gives the absorption of light in a medium, C is the concentration of the solution, and l is the length of the path that the light traveled (Cope, 1991). The absorbance of a solution which contains multiple compounds is given by adding the absorbance of each compound, given by Eq. 2 (Wray et al., 1988; Cope, 1991).

$$A=\sum_{i=1}^{n}\varepsilon C_{i}l=\left(\varepsilon_{1}C_{1}+\varepsilon_{2}C_{2}+\ldots +\varepsilon_{n}C_{n}\right)l$$ (2)

The Beer-Lambert Law is limited to non-scattering media or where scattering is negligible. In a tissue, or any multiple scattering media, the light takes a longer path, and this optical pathlength is accounted for in the Modified Beer-Lambert Law (Delpy et al., 1988). The optical pathlength has a scaling factor called differential pathlength factor (DPF), which quantifies the additional pathlength attributed to scattering. This law is given by Eq. 3 where with the inclusion of DPF and optode spacing given by d , there is another term, G , describing the tissue’s scattering coefficient with optode geometry. Due to the fact that G is unknown, the absolute chromophore concentration cannot be derived by Eq. 3 (Delpy et al., 1988; Bakker et al., 2012).

$$A=\text{log}\left(\frac{I}{I_{0}}\right)=\varepsilon \cdot C\cdot d\cdot \text{DPF}+G$$ (3)

With the assumption that the value of G is same for all chromophores in a medium, we can measure the relative concentration of chromophores by eliminating G using differential equation between two chromophores given by Eq. 4 where ΔA is the change in attenuation and ΔC is the change in chromophore concentration (Cope, 1991; Bakker et al., 2012).

ΔA=ε⋅ΔC⋅d⋅DPF (4)

Deoxyhemoglobin (HHb), Oxyhemoglobin (HbO), and Cytochrome C Oxidase (CCO) scatter and absorb NIR light differently. Thus, their measurements are performed with a NIRS device by calculating the change in attenuation for each absorber, also known as a chromophore, with different wavelengths. Using the Modified Beer-Lambert Law, the change in concentration for each chromophore can be solved with series of differential equations using multiple wavelengths. The Eq. 5 shows a system of equations that solves for concentrations of HbO $\left(\Delta C_{HbO}\right)$ and HHb $\left(\Delta C_{\text{HHb}}\right)$ using two wavelengths, $\lambda_{1}$ and $\lambda_{2}$ , given they have different molar extinction coefficients, $\varepsilon_{HbO}$ and $\varepsilon_{HHb}$ , (Cope, 1991; Scholkmann et al., 2014).

$$\left[\begin{array}{c} \text{Δ}C_{HbO}\\ \text{Δ}C_{HHb} \end{array}\right]=d^{-1}{\left[\begin{array}{cc} \varepsilon_{HbO,\lambda_{1}} & \varepsilon_{HbO,\lambda_{1}}\\ \varepsilon_{HHb,\lambda_{2}} & \varepsilon_{HHb,\lambda_{2}} \end{array}\right]}^{-1}\left[\begin{array}{c} \Delta {\text{A}}_{\lambda_{1}}/{\text{DPF}}_{\lambda_{1}}\\ \Delta A_{\lambda_{2}}/{\text{DPF}}_{\lambda_{2}} \end{array}\right]$$ (5)

### Different Near-Infrared Spectroscopy Techniques for Continuous Cerebral Physiology Measurement

The sub-sections below briefly outline different commercially available NIRS technology for continuous bedside/outpatient cerebral physiologic measurements. We touch on: fixed wavelength measures, spatially-resolved, frequency-resolved, time-resolved, and diffuse correlation spectroscopy (DCS) techniques. The provided descriptions are designed to be a primer. As such, we direct readers interested in more details to review the referenced literature. Supplementary Table S1 highlights the main NIRS technique categories for commercially available cerebral physiologic measurements.

### Common Continuous Fixed Wavelength Near-Infrared Spectroscopy Measurements

The main, naturally occurring chromophores of interest interrogated with NIRS are HbO, HHb, and CCO. While it is optically and computationally valid to assume that deoxy- and oxy-hemoglobin exist as distinct chromophores, the reality is that millions of hemoglobin molecules (contained in red blood cells) never exist in exclusively deoxy- or oxy-states. Nonetheless, NIRS measures the changes in concentration of these chromophores by calculating the change in attenuation for each of them. These compounds are able to be distinguished from one another because each one has its own unique absorbance spectrum and scatter NIR light in a unique way (Cope, 1991). HbO and HHb are of interest because they provide valuable clinical information regarding blood supply and oxygen transport for the tissues of interest (Davies et al., 2015). Unique information on the status of intracellular oxygenation is provided by monitoring the redox state of CCO; however, CCO is difficult to calculate with only a few wavelengths and typically requires hyperspectral NIRS systems using numerous wavelengths across the entire NIR spectrum (Bale et al., 2016). Along with the measurement of the concentration changes of the above three chromophores, spatially resolved NIRS such as NIRO-300 can derive Tissue Oxygenation Index (TOI) and Total Hemoglobin Index (THI) (Davies et al., 2015). TOI is the ratio of HbO to total tissue hemoglobin (HbT) (Suzuki et al., 1999). It has been shown that the measurement of the TOI reflects intracranial brain tissue oxygenation to a high degree of sensitivity and specificity as compared to intracranial changes in transcranial Dopper based middle cerebral aretery cerebral blood flow velocity in adults (Al-Rawi et al., 2001). THI provides a surrogate for the total tissue hemoglobin of the measured tissue. Since the exact total volume of the tissue, observed through absorption of emitted light, is not known, it is expressed as an arbitrary unit. Regional cerebral oxygen saturation (rSO_2_) is another measure for cerebral tissue oxygenation, like TOI, but is reported by INVOS devices. Each manufacturer uses different proprietary methodologies and assumptions to calculate their measure of cerebral tissue oxygenation and as a result, the interchangeability of TOI and rSO_2_ still is unclear (Thavasothy et al., 2002). Readers interested in a more detailed description of continuous wave NIRS technology are directed to a recent review by Scholkmann and collogues (Scholkmann et al., 2014).

### Spatially Resolved Near-Infrared Spectroscopy

**FIGURE 1:**
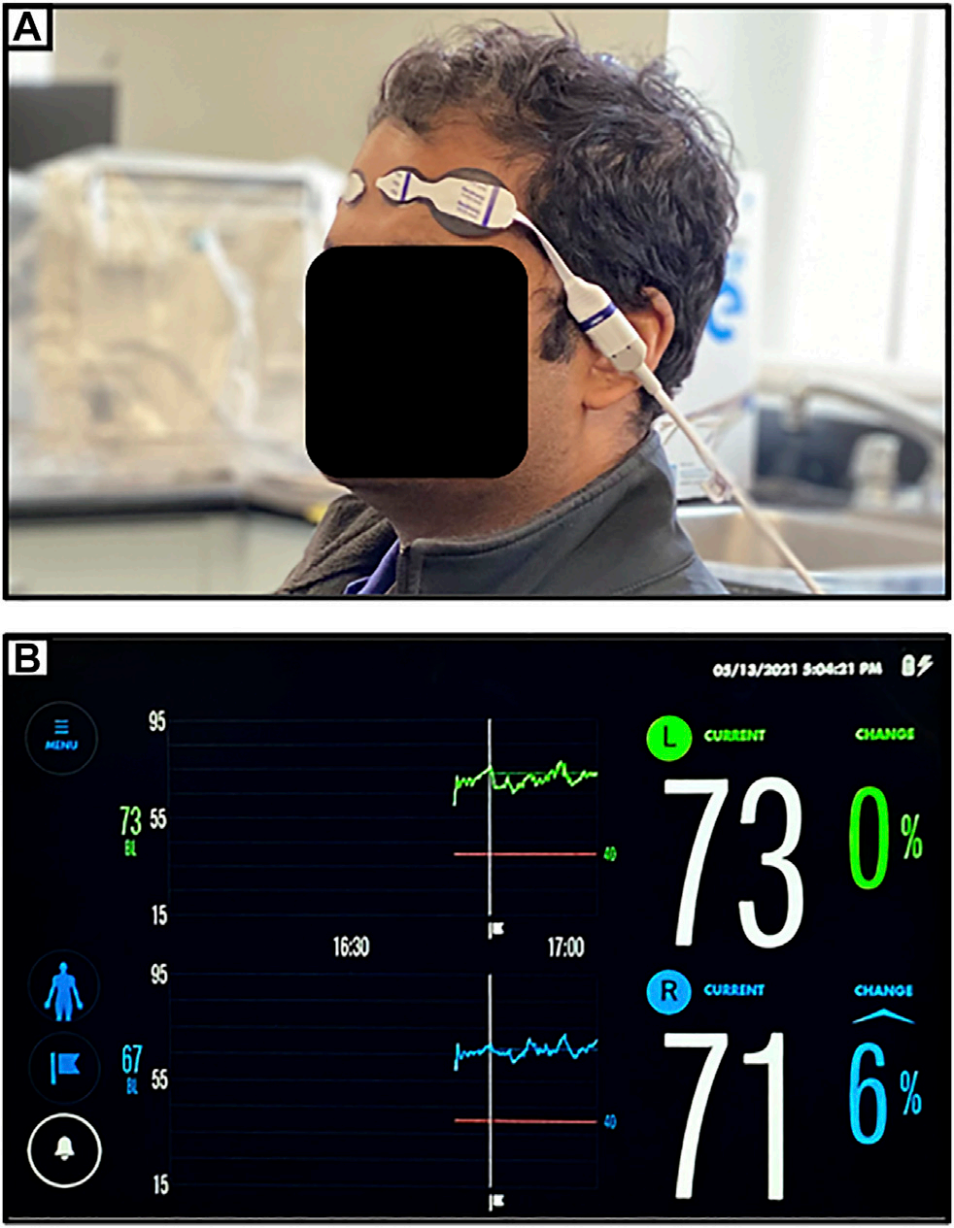
**(A)** Setup for near-infrared spectroscopic (NIRS) monitoring of bilateral frontal cerebral oxygenation (as demonstrated on first author). **(B)** Typical device readout of regional cerebral oxygen saturation (rSO_2_) from NIRS monitoring of bilateral frontal cerebral oxygenation.

The spatially resolved NIRS uses multiple light detectors (two or more) placed together in each probe and at a distance of a couple of centimeters from the NIR light source. With multiple light detectors, a purer cerebral measure can be obtained by eliminating the extracranial circulation of the scalp, recorded from short path light detector, from the long path light detectors that are able to penetrate deeper to the brain tissue. The attenuation of NIR light at different wavelengths is measured using multiple light detectors on the illuminated tissue to derive relative concentrations of HHb and HbO, and hence be able to estimate mean tissue hemoglobin saturation (Matcher et al., 1995). Commercially available spatially resolved NIRS are NIRO-300 which is available in two channels (Suzuki et al., 1999), and INVOS, which is available in two or four channels (Thavasothy et al., 2002; Hessel et al., 2014). Spatially resolved NIRS is currently used for bedside monitoring of TBI patients. The technical limitation of spatially resolved NIRS is that it cannot quantify the scattering parameters in its calculation, giving the relative concentrations of chromophores instead of the absolute concentrations. Figure 1 provides an example of a commercially available spatially resolved NIRS platform, applied to the first author of this manuscript.

### Frequency-Resolved Near-Infrared Spectroscopy

The frequency-resolved NIRS generally uses a laser diode or LED as the light source, which is intensity-modulated at radio frequencies. The measurement is made of the detected light intensity along with its phase shift and modulation depth with respect to the input light, which provides information on the scattering properties of the medium (Lakowicz and Berndt, 1990). These measurements can provide the absolute quantification of hemodynamics. A linear relationship exists between frequency and phase shift up to 200 MHz for the light transmitted through tissues. This relationship is not present with higher frequencies (Arridge et al., 1992). This type of NIRS is widely used in clinical frequency-resolved systems are also known as frequency domain, intensity-modulated or phase-resolved systems. A commercially available frequency-resolved NIRS is FORE-SIGHT which has only one channel and measures regional cerebral tissue oxygenation (cStO_2_) (Hessel et al., 2014). The technical limitation of frequency-resolved NIRS is that if only one frequency is used, less information about the tissue will be provided.

### Time-Resolved Near-Infrared Spectroscopy

The time-resolved NIRS inputs light in tissues using a picosecond laser source to generate ultrashort pulses, and the intensity of the emergent light from the tissue is detected as the temporal point spread function (TPSF) with picosecond resolution (Chance et al., 1988; Delpy et al., 1988). The ultrafast detector used in this type of system is the streak camera with the advantage of high temporal resolution or a time-correlated photon counting system that can provide a wide dynamic range while using cheaper components. Time-resolved systems are also known as time-domain or time-of-flight systems. They contain information about changes in different depths of light absorption (Weigl et al., 2016), whereby photons from superficial paths will arrive earlier than photons from deep tissue paths. Commercially available time-resolved NIRS is TRS-10 which has only one channel and measures oxygen saturation (SO_2_) (Oda et al., 1999). The technical limitations of time-resolved NIRS using the streak camera are the limited dynamic range, higher cost, and large size, making it difficult to use in a clinical environment. Moreover, the system uses a time-correlated photon counting system which is limited by its low speed. For those readers interested in a more detailed reviews of this method we direct them to recent reviews on time-resolved NIRS (Torricelli et al., 2014; Lange and Tachtsidis, 2019).

### Diffuse Correlation Spectroscopy Integrated With Near-Infrared Spectroscopy

There is an emerging optical technique called diffuse correlation spectroscopy (DCS), which can measure blood flow by using temporal fluctuations of the reflected NIR light to quantify the blood flow and provide a direct measure of cerebral blood flow (CBF) (Durduran and Yodh, 2014). The fluctuations in the reflected NIRS light is caused by the light being scattered by moving red blood cells and based on photon arrival times detected, these fluctuations are quantified by intensity temporal autocorrelation function (Buckley et al., 2014; Durduran and Yodh, 2014). It has been demonstrated that integrating frequency-resolved NIRS and DCS into an instrument, known as MetaOx, is convenient for continuous data collection, and the combination of oximetry and flow measures can quantify cerebral tissue oxygen metabolic rate (CMRO_2_) (Carp et al., 2017). Also, by combining DCS with Time-resolved NIRS, it has been shown to measure absolute CMRO_2_ and verified against values of CMRO_2_ derived from cerebral venous blood samples (Verdecchia et al., 2013). So, DCS can be paired with any NIRS system to determine information about the metabolic rate of oxygen. The technical limitations of DCS is that it is sensitive to superficial cortical regions, more so in adults than in neonates, with the setup of having the source-detector separations of 2–3 cm. Increasing the penetration depth to detect cerebral signals in adults requires larger source-detector distance which is challenging because it leads to low signal-to-noise ratio (SNR) since DCS requires single-mode detection fibers. Grouping multiple detector fibers is an expensive option because it requires additional photon counting detectors to produce autocorrelation curves which can then be averaged to derive a single curve (Buckley et al., 2014). With low SNR and the presence of hair and/or dark skin, the acquisition times can increase along with reducing temporal resolution (Buckley et al., 2014; Durduran and Yodh, 2014).

## Near-Infrared Spectroscopy in the Clinical Managment of Traumatic Brain Injury

### Near-Infrared Spectroscopy in Pre-hospital/Emergency Department Management of Traumatic Brain Injury

Currently, in the setting of TBI, severity of injury is primary evaluated by the patient’s clinical exam (Teasdale and Jennett, 1974) and computed tomography (CT) imaging features (Marshall et al., 1991; Maas et al., 2005). As a result, these two factors play a large role in triaging of TBI patients. With the limited portability of CT imaging machines, the assessment of TBI patients, in the prehospital setting, has largely been limited to clinical examination. This has left a void that may be ideally filled by NIRS. Given their non-invasive nature, portability, relative low cost, and ease of use, NIRS devices have been an attractive modality for the pre-hospital and emergency department assessment of TBI patients. Feasibility studies for their use in the pre-hospital setting have already been conducted and have found their use practical (Weatherall et al., 2012). Moreover, the availability of handheld form factors has made NIRS a technology ideal for application in this setting (Leon-Carrion et al., 2010).

The most promising application has been the detection of intracranial hemorrhage in TBI patients prior to them having undergone CT imaging. Gopinath et al. (1993) described an early study of 42 patients with severe TBI. In this cohort, of the 40 patients with intracranial hemorrhages as identified by CT, all had increased absorption of 760 nm NIR light on the side with the hematoma. It was noted that the difference in optical density (OD) between hemispheres was more pronounced for epidural hematomas (EDH, 0.99 +/− 0.30) and subdural hematomas (SDH, 0.87 +/−0.31) than for intracerebral hemorrhages (ICH, 0.41 +/−0.11). They also noted that the difference in hemispheric OD was minimal in the setting of diffuse axonal injury (DAI) and at a similar level to that found in healthy controls (Gopinath et al., 1993).

This work was expanded on by Robertson et al. (1997) in a study of 305 patients with TBI. Similarly, this study examined hemispheric differences in OD as measured by 760 nm NIRS. Of the 305 patients, 191 presented with an intracranial hemorrhage as detected by CT imaging. As with the study by Gopinath et al. (1993), detection by NIRS of EDH, SDH, and ICH was better than that for DAI. The hemispheric difference in OD was more pronounced in those with an intracranial hemorrhage than that found in a cohort of healthy controls (0.78 vs. 0.03, *p* < 0.001). Patients with DAI had hemispheric differences in OD similar to the healthy control cohort. Interestingly, the size of EDH and SDH was significantly related to the hemispheric difference in OD (*R* ^2^ = 0.77, *p* < 0.001 and *R* ^2^ = 0.55, *p* < 0.001, respectively). Of note, in this study, one patient with a deep basal ganglia ICH had no detectable hemispheric difference in OD, highlighting the depth of penetration as a limitation to this method (Robertson et al., 1997).

The increased OD in the hemisphere with post-traumatic hemorrhage is attributable to the increased absorbance of acute extravascular blood, which has a higher concentration of hemoglobin than normal brain tissue (Zhang et al., 2000). This dependence on acute clot may explain the failure to identify chronic SDH utilizing hemispheric difference in OD in later studies (Kahraman et al., 2006). The sensitivity and specificity of NIRS for the detection of post-traumatic intracranial hemorrhage is between 68.7–90.5 and 77.7–95.5%, respectively, as determined in multiple studies involving 35 to 365 TBI patients (Kessel et al., 2007; Ghalenoui et al., 2008; Leon-Carrion et al., 2010; Robertson et al., 2010).

This method does have its limitations, however. Most notably, by using the contralateral side as a control, the ability to identify bilateral hemorrhages is eliminated. Additionally, as noted previously, the depth of interrogation is limited and so deep hemorrhages may go undetected. Finally, scalp hematomas obscure readings and can result in both false positives and false negatives (Robertson et al., 1997).

In a more recent study by Kontojannis et al. (2019) 205 trauma patients were screened, using a handheld NIRS device, for traumatic intracranial hematomas upon presentation to the emergency department. The sensitivity and specificity were found to be 75 and 50.43% respectively with a negative predictive value of 72.84% and a positive predictive value of 53.23%. Notably, for hematomas where the blood volume was >3.5 ml the negative predictive value was 93.9%, indicating a possible role for NIRS in ruling out surgically relevant intracranial lesions (Kontojannis et al., 2019). This could be a valuable tool when trying to determine if a patient will need to be transported to a hospital with neurosurgical expertise.

The role of NIRS in the pre-hospital and emergency department setting for TBI patients is not to replace CT imaging but to act as a possible means of triaging patients beyond clinical exam. In fact, the heterogeneity of injury in TBI can greatly impact signal quality, it is essential to review CT imaging to insure appropriate placement of the optodes. Additionally, lateral placement, when placing optodes in the frontal region is important to avoid variations in the frontal sinus.

### Near-Infrared Spectroscopy in Intraoperative Monitoring of Traumatic Brain Injury Patients

The monitoring of cerebral physiology by NIRS during intracranial surgery following TBI has not become commonplace. The most likely reason is that the scalp, where most devices are placed, is reflected during trauma craniotomies or craniectomies. There are descriptions of subdural NIRS devices that are placed on the cortical surface, however data surrounding their use is limited (Keller et al., 2003).

The role of NIRS in intraoperative cerebral monitoring can be found in the acute surgical management of extracranial injury following TBI. Moderate and severe TBI is often accompanied by extracranial injuries that require surgical intervention. Currently, invasive ICP monitoring is typically the only form of cerebral monitoring in this setting, with other modalities not frequently adopted (Carney et al., 2017). Given the invasive nature of the placement of ICP monitors, they require neurosurgical or neurocritical care expertise. This limits their use to specialist centres. NIRS optodes are non-invasive and easily applied, making them an appealing alternative to more invasive means of cerebral monitoring in the intraoperative setting.

NIRS has been demonstrated in the non-trauma setting, have been explored as a means of determining the personalized effects of sedative agents. In a study by Curtin et al. (2014), 41 patients undergoing colonoscopy were monitored with functional NIRS as propofol sedation was administered. They found that HbO levels reduced in the dorsolateral prefrontal cortex in response to bolus administration of propofol while blood oxygen saturation, heart rate, blood pressure and ETCO_2_ remained constant (Curtin et al., 2014). Taking this concept further, Hernandez-Meza et al. developed a functional NIRS based machine learning classifier to classify, in real-time, maintenance and emergence states in 19 patients undergoing various surgical procedures with sevoflurane anesthetic. They found that their NIRS based system was able tot detect emergence prior to bispectral index (BIS; Hernandez-Meza et al., 2017). These studies point to a possible role for NIRS in the tailored titration of sedation in the acute setting.

There are a number of studies demonstrating the appeal of NIRS for the intraoperative monitoring of cerebral oxygenation. In a report by Plachky et al. (2004), they monitored 16 patients undergoing orthotopic liver transplantation. Of the 16 patients, 8 developed decreased rSO_2_ following clamping of the vena cava. These decreases were associated with increases in serum biomarkers of neuronal damage including neuron-specific enolase (NSE) and S-100 (*R* ^2^ = 0.57, *p* < 0.05 and *R* ^2^ = 0.52, *p* < 0.05, respectively). Notably, increases in these biomarkers were not associated with changes in cardiac output (Plachky et al., 2004). This indicates the sensitivity of NIRS at detecting intraoperative cerebral injury beyond typical physiologic monitoring methods.

In the field of cardiac anesthesia, NIRS technology has been extensively explored as a means of monitoring cerebral physiology during surgery. Zavriyevet al. (2021) have recently reported on the tandem use of DCS and frequency resolved NIRS to measure CBF and CMRO_2_ concurrently in 12 patients undergoing elective cardiac surgery with hypothermic circulatory arrest. The found that the information provided by these devices was able to help achieve patient specific optimal brain perfusion (Zavriyev et al., 2021). In a similar study, Rajaram et al. (2020) explored the hybrid NIRS system that combined DCS and fixed wavelength NIRS to examine CBF and the cerebral oxidation state of CCO in 10 patients undergoing cardiac surgery with cardiopulmonary bypass. Once again, they found that this intraoperative NIRS based multimodal monitoring aided in the real-time assessment of patient specific cerebral hemodynamics (Rajaram et al., 2020).

Functional outcomes have also been examined in relation to rSO_2_ as measured by NIRS. In a study by Casati et al., a cohort of 122 elderly patients undergoing abdominal surgery were monitored by NIRS with 56 of the patients randomized to the treatment of rSO_2_ values less than 75%, while 66 of the patients were randomized to observation only. Those in the treatment group were found to have significantly higher Mini-Mental State Examination (MMSE) scores on postoperative day number 7 [26 (25–30) vs. 28 (26–30), *p* = 0.02]. There was also a correlation between time with an rSO_2_ < 75% and MMSE score on postoperative day number 7 (*R* ^2^ = 0.26, *p* = 0.01) as well as length of hospital stay (*R* ^2^ = 0.40, *p* = 0.001; Casati et al., 2005). This highlights that not only is rSO_2_ an important physiologic parameter but also that targeted management may improve functional outcomes. Unfortunately, no similar study has been performed in TBI patients undergoing extracranial surgery.

The role of NIRS for intraoperative monitoring is still unclear, with the most recent Cochrane review on the subject finding that only low-quality evidence exists for its use (Yu et al., 2018). Further multicenter randomized trials in the setting of TBI are required before NIRS can replace or even augment ICP monitoring in the intraoperative setting acutely after TBI.

### Near-Infrared Spectroscopy in Neurocritical Care of Traumatic Brain Injury Patients

**FIGURE 2:**
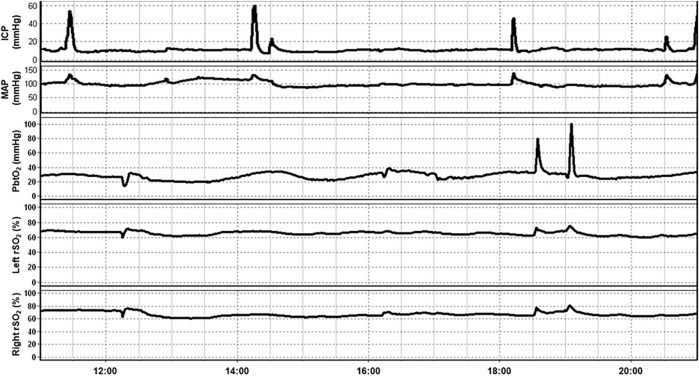
An example of continuous 10-h recording of mean arterial pressure (MAP), intracranial pressure (ICP), brain tissue oxygenation (PbtO_2_), and regional cerebral oxygen saturation (rSO_2_) in a traumatic brain injury patient. Note that fluctuation in rSO_2_ tends to occur prior to associated fluctuations in PbtO_2_. Data taken from previously published and approved studies (University of Manitoba REB: H2017:181, H2017:188 and H2020:118; Bernard et al., 2020; Froese et al., 2020b, 2020a, 2021; Thelin et al., 2020).

Much of the work done exploring the role of NIRS in the management of TBI patients has been conducted in the neurocritical care phase of management. In this section, we explore the various applications of NIRS in this setting. Figure 2 provides an example of a multimodal monitoring data stream for a severe TBI patient. The raw bilateral non-invasive NIRS-based rSO_2_ measures are measured concurrently with invasive ICP, PbtO_2_ and mean arterial pressure (MAP) monitoring from our existing, approved (University of Manitoba REB: H2017:181, H2017:188, H2020:118) and previously published database work in moderate/severe TBI (Froese et al., 2020a, Froese et al., 2020b; Froese et al., 2021; Bernard et al., 2020; Thelin et al., 2020).

### Delayed Hematoma Formation

One of the earliest explored applications of NIRS in the neurocritical care setting following TBI was for the detection of delayed hematomas. Gopinath et al. (1995) serially examined 167 moderate/severe TBI patients using NIRS in the 3 days following injury to detect the formation of delayed intracranial hematomas. In particular, they measured the hemispheric difference in OD at 760 nm. During this period, 27 of the patients developed delayed hematomas as detected by CT imaging. Of these 27 patients, 24 patients had an increase of their hemispheric difference in OD that appeared prior to increases in ICP or a change in their neurologic exam (Gopinath et al., 1995). In a follow-up study, the cohort was expanded to 305 patients with TBI, of which 59 developed delayed hematomas. Notably, an increase in the hemispheric difference in OD of greater than 0.1 was observed in 55 of the 59 patients that developed delayed hematomas. Once again, this difference was present prior to an increase in ICP or a change in neurologic examination (Robertson et al., 1997).

These studies indicate that NIRS may have a role in the early detection of delayed hematomas following TBI. While CT imaging is still the “gold standard” for detection and required for operative planning, NIRS may allow for early detection before neurologic decline or elevations in ICP. This early detection may allow for the more targeted use of delayed CT imaging following TBI. This is especially important in the neurocritical care setting where neurological examination may be limited and where the transportation of patients to the CT scanner is cumbersome and not without risk.

### Cerebral Oxygenation

There is currently no “gold standard” for the measurement of cerebral oxygenation, however two invasive methods are commonly found in neurocritical care units. Jugular venous bulb oximetry (SjVO_2_) is a method by which measurements of oxygen tension are obtained from samplings of the venous blood collected from the jugular bulb. This method is thought to reflect a global hemispheric measurement of cerebral oxygenation (Macmillan and Andrews, 2000). More recently, brain tissue oxygenation (PbtO_2_) monitoring has become popular in which a Clarke electrode is placed into the brain parenchyma directly. This method provides a measure of regional extracellular oxygen content, which is thought to reflect cerebral oxygenation (De Georgia, 2015). It should be noted that SjVO_2_, PbtO_2_, and NIRS all measure different components of cerebral oxygen content but given that there is no “gold standard” these modalities have been compared in the setting of TBI.

In an early study of 9 patients with severe TBI, rSO_2_ was found to not reflect changes in SjVO_2_ following ABP manipulation by vasopressors or manipulation of CO_2_ (Ter Minassian et al., 1999). This lack of correlation may not, however, reflect a weakness in NIRS as a previous study of 14 severe TBI patients found that NIRS was more sensitive to changes in CPP, accompanied by changes in cerebral blood flow velocity (CBFV) and cortical perfusion than jugular venous bulb oximetry (Kirkpatrick et al., 1995).

As can be seen in Figure 2, a temporal correlation can frequently be seen between non-invasively measured rSO_2_ and invasively measured PbtO_2_ which has sparked investigations comparing these modalities. In fact, the relationship between NIRS based measurements of cerebral oxygenation and PbtO_2_ has been examined on multiple occasions. In a cohort of 10 severe TBI patients, 137 events were identified where PbtO_2_ changed >10% from baseline. A corresponding change in HbO_2_, as measured by NIRS, was observed 77.4% of the time with a good correlation between the two measurements (*R* = 0.73, no *p*-value reported; Holzschuh et al., 1997). Similarly, in a study of 19 severe TBI patients and 12 aneurysmal subarachnoid hemorrhage (SAH) patients, there was a good correlation between rSO_2_ and PbtO_2_ values during episodes of induced hyperoxia (*R* = 0.67, *p* < 0.01; Büchner et al., 2000). A similar study including 10 TBI patients and 3 SAH patients found a high degree of correlation between both modalities in 80% of datasets (Rothoerl et al., 2002).

In a study of 22 severe TBI patients, Leal-Noval et al. (2010) found that NIRS could detect moderate and severe hypoxia as defined by PbtO_2_ < 15 mmHg (AUC 0.6, 95%CI 0.60–0.63, *p* < 0.0001) and PbtO_2_ < 12 mmHg (AUC 0.82, 95%CI 0.80–0.82, *p* < 0.0001), respectively. Newer modalities of NIRS, such as ultrasound tagged NIRS (UT-NIRS) and frequency-resolved NIRS have failed to correlate well with PbtO_2_ measurements in cohorts of TBI patients (Rosenthal et al., 2014; Davies et al., 2019).

While the authors of these studies interpreted the lack of ability of NIRS to precisely match PbtO_2_ as a failure of NIRS, it should be noted that PbtO_2_ is not held to be a “gold standard.” In fact, Budohoski et al. found that in a study of 42 TBI patients, PbtO_2_ changes were significantly slower than those identified in NIRS based metrics following changes in ABP [39.6s (IQR 16.4–66.0) vs. 10.9s (IQR -5.9–39.6), *p* < 0.001] and ICP [22.9s (IQR 11.0–53.0) vs. 7.1s (IQR -8.8–195), *p* < 0.001; Budohoski et al., 2012]. NIRS was also found to better detect changes associated with induced normobaric hyperoxia in a cohort of 8 severe TBI patients, as compared to both PbtO_2_ and SjVO_2_ (McLeod et al., 2003).

NIRS measures of cerebral oxygenation have been associated with functional outcomes following TBI. In a study of 18 severe TBI patients, episodes of cerebral hypoxia, as defined by an rSO_2_ < 60%, were significantly more common in those that died than those that survived (36.1 vs. 16.3 OR 2.9, *p* < 0.0001; Dunham et al., 2004). Esnault et al. (2015) also found a similar result in their cohort of 8 neurocritical care patients, 4 with TBI, where those with good outcomes had significantly better rSO_2_ values than those with poor outcomes [78% (73–81) vs. 65% (55–71), *p* < 0.0001] based on Glasgow Outcome Scale (GOS) at discharge from ICU. A larger study of 61 TBI patients found rSO_2_ to be a better predictor of in-hospital mortality than admission GCS, blood sugar, or hemoglobin. Those that survived were also noted to have higher NIRS values in the time between admission and undergoing surgery (75.4% +/− 9.8 vs. 71.0% +/− 20.5%, *p* = 0.013) and at 1 h after admission to ICU (74.7%+/− 1.5 vs 61.9% +/− 19.4%, *p* = 0.029; Vilkė et al., 2014).

NIRS can be seen as a non-invasive alternative method of measuring cerebral oxygenation in the neurocritical care setting following TBI. While some early evidence indicates its reliability and prognostic utility, as with other invasive modalities, its clinical relevance is not fully defined.

### Cerebral Oxygen Metabolism

The versatility of NIRS allows for various chromophores to be examined, and as a result, the evaluation of oxygen metabolism can be interrogated through the measurement of CCO. In a 2007 paper, Tisdall et al. (2008) conducted a study where normobaric hyperoxia was induced in 8 adults, ventilated TBI patients while oxidized CCO concentrations were measured with NIRS. Additionally, PbtO_2_ was measured, while microdialysis was used to measure lactate to pyruvate ratios (LPR). Increased oxidation of CCO, as measured by NIRS, was correlated with increase in PbtO_2_ (R = 0.57, *p* = 0.005) and decreases in LPR (R = -0.53, *p* = 0.006; Tisdall et al., 2008). A later study confirmed that these findings were not simply due to changes in optical scattering or pathlength (Ghosh et al., 2013). Hyperoxia was also shown to increase concentrations of oxidized CCO in a study of 16 acute brain injury patients, 7 of whom had experienced TBI (Ghosh et al., 2017). These studies indicate that NIRS may provide a non-invasive alternative to microdialysis with a better spatial and temporal resolution, however, significantly more work is needed to clarify its role.

### Cerebral Blood Flow

Given that NIRS can detect changes in hemoglobin concentration in tissue, it has been evaluated as a surrogate for detecting changes in CBF following TBI; notably, however, changes in concentration are not equivalent to changes in blood flow, and should not be conflated. In a study of 14 ventilated TBI patients, NIRS was able to identify 37 out of 38 events of altered cortical perfusion as detected by laser Doppler flowmetry secondary to changes in CPP (Kirkpatrick et al., 1995). Smaller studies have also shown a similar relationship between rSO_2_ and CPP (Dunham et al., 2002).

More recent studies have found that rSO_2_ does not correlate well with CBF as measured by Xenon CT. In a small cohort of 7 neurocritical care patients, 2 of whom had a TBI, Kim et al. (2010) and colleagues found that changes in HgO and total hemoglobin were not significantly correlated with CBF as measured by Xenon CT. Shafer et al. (2011) found similar results in a study of 22 patients, 3 of whom had TBI. It is clear that the relationship between rSO_2_ and CBF is more complex as has been demonstrated in a recent case report of persistent normal rSO_2_ during prolonged cardiac arrest (Maillard et al., 2019). This might possibly be explained by reduced metabolic demand resulting in the maintenance of cerebral oxygen levels in the absence of CBF. A more nuanced approach, beyond interpreting rSO_2_ values as a surrogate for CBF, must be taken when evaluating spatially resolved NIRS measurements.

While spactially resolved NIRS modalities are limited in their assessment of CBF, time-resolved methods utilizing indocyanine green (ICG) bolus tracing show promise in the setting of TBI (Weigl et al., 2014). Additionally, in the previously described study by Kim et al. (2010), measurements of CBF by DCS performed better than the spatially resolved modality and correlated well with measurements made by Xenon CT (R = 0.73, *p* = 0.010). In a healthy population of 10 subjects ICG contrast enhanced NIRS was validated as a measure of CBF against magnetic resonance imaging (MRI) perfusion (Milej et al., 2020). These contrast enhanced NIRS methods are more technically complex but are still substantially less cumbersome than conventional assessments of CBF such as Xenon CT and MRI perfusion.

### Cerebrovascular Reactivity/Cerebral Autoregulation

Cerebral autoregulation (CA) is the physiologic process in which CBF is maintained relatively constant over a range of ABPs. The mechanism by which this process occurs, cerebrovascular reactivity, is through the constriction and dilation of cerebral arterioles to maintain a constant blood flow (Lassen, 1959). Dysfunctional CA following TBI is thought to contribute to secondary injury and worse functional outcomes (Donnelly et al., 2019). Continuous bedside monitoring of cerebrovascular reactivity following TBI has garnered interest as it has been found to be associated with outcomes following injury (Zeiler et al., 2019). The pressure reactivity index (PRx) utilizes ICP as a surrogate for cerebral blood volume (CBV), which is in turn related to CBF and quantifies how it moves with changes in ABP through the use of a continuously updating Pearson correlation coefficient between the two variables (Czosnyka et al., 1997). PRx has a growing body of evidence and has become a “gold standard” for continuous bedside assessment of cerebrovascular reactivity (Zweifel et al., 2008). It is, however, limited by its dependence on invasively measured ICP.

NIRS-based metrics of cerebral oxygenation have been found to correlate strongly with CBV, as measured by CT perfusion, in a population of 25 TBI patients (R = 0.9, *p* < 0.000001; Trofimov et al., 2016). This finding has led to the development of various NIRS-based indices of cerebrovascular reactivity (THx, TOx, and COx) that substitute ICP with NIRS-based metrics (THI, TOI, and rSO_2,_ respectively). NIRS-based indices, like PRx, have been validated in animal models to accurately detect the lower limit of CA (Lee et al., 2009). In a mixed cohort of 150 patients, 40 of whom had severe TBI, Smielewski et al. (2010) found that THx, TOx, and COx correlated well with PRx and TCD-based indices.

In a group of 40 TBI patients, Zweifel et al. (2010) found that THx and PRx correlated well with averaged individual recordings (R = 0.49, *p* < 0.0001) and across patients (R = 0.56, *p* = 0.0002). Notably, THx showed a strong ability to detect disrupted cerebrovascular reactivity, as defined as a PRx >0.3 (AUC 0.772, 95%CI 0.59–0.96, *p* = 0.016). In a 2015 study of 27 sedated TBI patients, PRx was found to correlate well with both THx (R = 0.63, *p* < 0.0001) and TOx (R = 0.40, *p* = 0.04); however, wide limits of agreement were found between these indices by Bland-Altman analysis (Highton et al., 2015).

**FIGURE 3:**
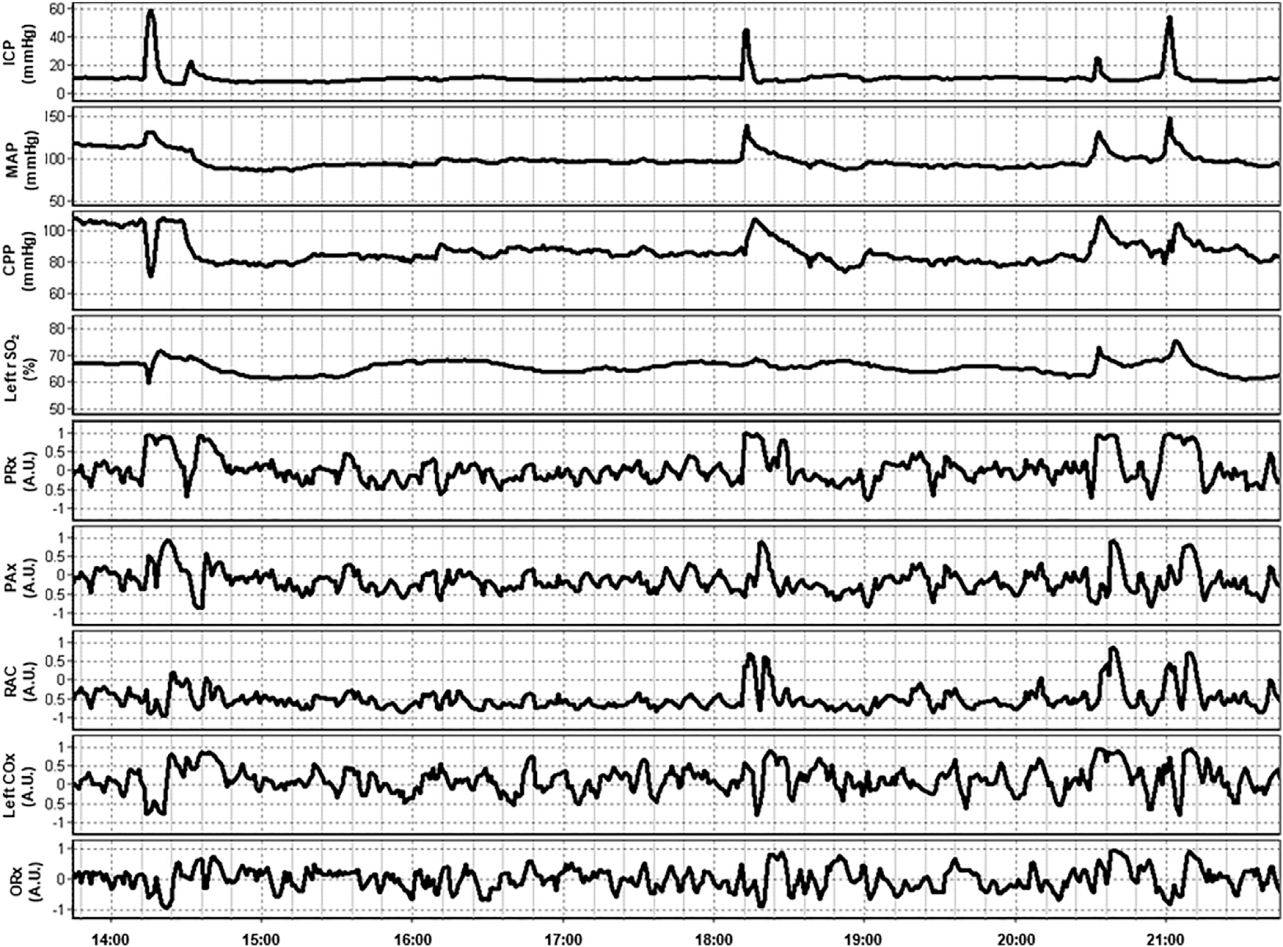
An example of continuous 8-h recordings of intracranial pressure (ICP), mean arterial pressure (MAP), cerebral perfusion pressure (CPP), and regional cerebral oxygen saturation (rSO_2_) in a traumatic brain injury patient. Also demonstrated are continuous ICP-based (PRx, PAx, and RAC), near-infrared spectroscopy (NIRS) based (COx), and brain tissue oxygenation (PbtO_2_) based (ORx) indices of cerebrovascular reactivity. Data taken from previously published and approved studies (University of Manitoba REB: H2017:181, H2017:188 and H2020:118; Bernard et al., 2020; Froese et al., 2020b; Froese et al., 2020a; Froese et al., 2021; Thelin et al., 2020).

In a multi-modal analysis of various ICP-, TCD- and NIRS-based indices of cerebrovascular reactivity by Zeiler et al. in 37 TBI patients, THx and TOx correlated with each other and ICP-based and TCD-based indices. Additionally, clustering analysis found these NIRS-based indices to co-cluster with ICP-based indices, such as PRx. NIRS-based indices failed to significantly discriminate between the patients with good and bad outcomes following TBI. Moreover, they were unable to differentiate between survivors and those who died. This lack of ability may, however, be attributable to the small sample size (Zeiler et al., 2017). Figure 3 provides an example of continuous NIRS, ICP, and PbtO_2_ based cerebrovascular reactivity. This demonstrates how these indices can both be generated in a continuous fashion in real time and can fluctuate through the course in hospital.

There is strong evidence that NIRS-based indices of cerebrovascular reactivity may be a viable alternative to ICP derived indices such as PRx. These less invasive indices also have the added advantage of evaluating hemispheric differences in cerebrovascular reactivity. Large comparative prospective trials need to be conducted before their role can be fully understood.

### Near-Infrared Spectroscopy in the Rehabilitation and Outpatient Follow-Up of Traumatic Brain Injury Patients

The non-invasive nature of NIRS allows for the evaluation of metrics in TBI patients beyond the acute phase of injury and into the rehabilitation and outpatient phases of care. NIRS has been described in the use of monitoring the recovery of cerebral oxygenation during the rehabilitation of TBI patients and may be a means of tracking improvements in motor and cognitive function (Bhambhani et al., 2006; Hashimoto et al., 2008). Additionally, entirely non-invasive methods of monitoring cerebrovascular reactivity have been described and may provide a method of tracking the recovery of CA following TBI in the outpatient setting (Gomez et al., 2020b; Gomez et al., 2020a).

**FIGURE 4:**
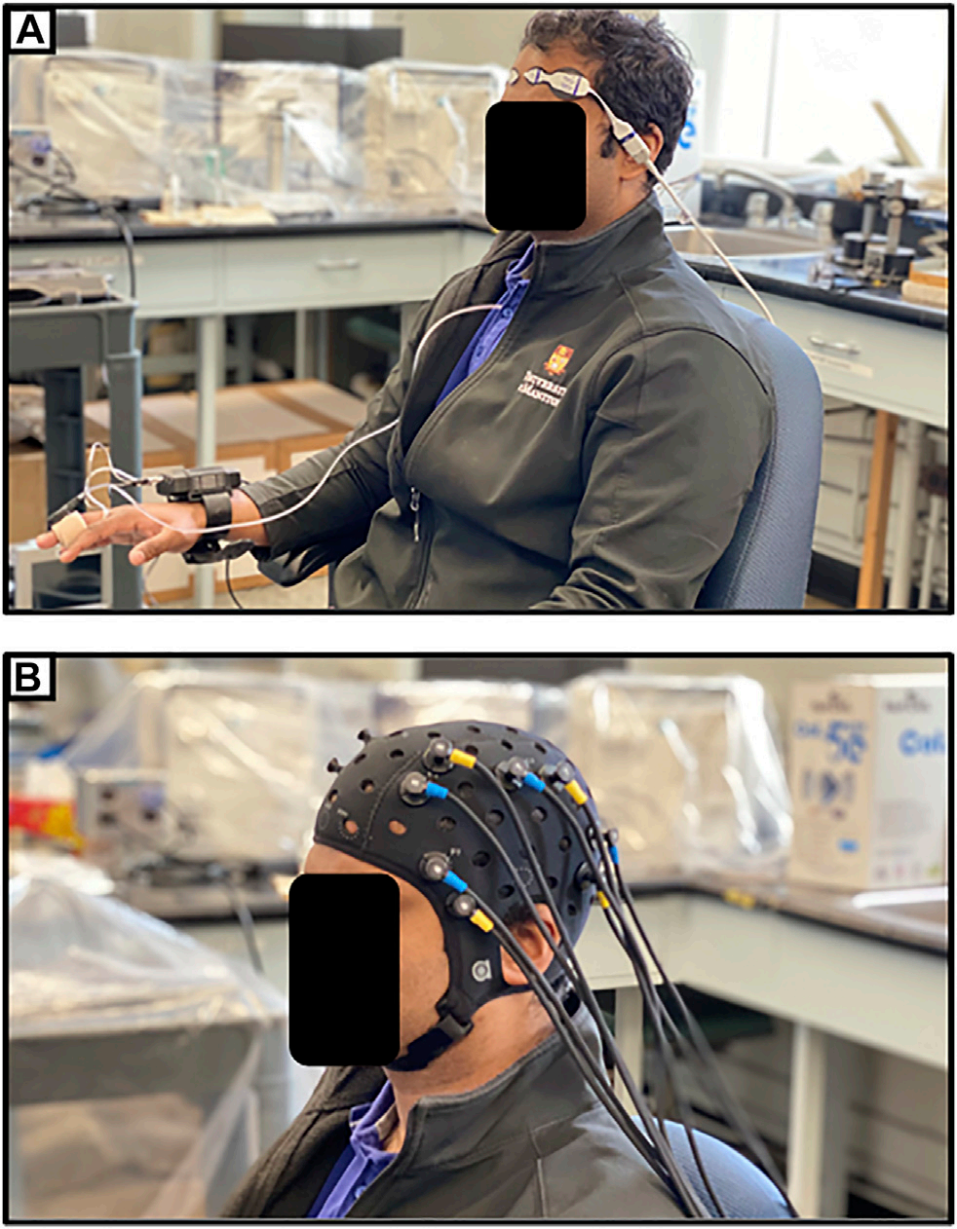
**(A)** Entirely non-invasive setup for measuring cerebrovascular reactivity using near-infrared spectroscopy (NIRS) and continuous non-invasive blood pressure monitoring (NIBP). **(B)** Typical multi-channel functional NIRS (fNIRS) device setup. Both as demonstrated on first author.

One promising application of NIRS in the post-acute setting following TBI is through functional NIRS (fNIRS), which operates similarly to functional MRI (fMRI). High-density NIRS based imaging arrays have been shown to map higher-order, distributed brain function without the ionizing radiation or strong magnetic field found in positron emission tomography (PET) or fMRI (Eggebrecht et al., 2014). Hibino et al. (2013) described a study in which 9 patients with cognitive impairment following TBI and 47 healthy controls were examined using fNIRS. Their device utilized 47 NIRS channels, measuring changes in HbO in regions of the frontal and temporal lobes. They noted that the post-TBI cohort had different regions of functional activation than the healthy controls while performing the same tasks (Hibino et al., 2013). Similar studies have found differences in activity during verbal and memory tasks (Kontos et al., 2014; Rodriguez Merzagora et al., 2014). One recent case report has even reported fNIRS as part of a brain-computer interface enabling a locked-in patient to communicate (Abdalmalak et al., 2017). Eventually, fNIRS may provide insights into how the brain functionally recovers following TBI, and its ease of use may allow for it to be a clinically useful means of tracking recovery. Figure 4A provides an example of the setup for continuous non-invasive cerebrovascular reactivity monitoring at the bedside or in an outpatient setting, as demonstrated by the first author in this photo. Figure 4B displays an fNIRS device that provides high-frequency HbO and Hb outputs at numerous channels with customizable layouts, as demonstrated by the first author in this photo.

### Future Directions for Near-Infrared Spectroscopy in Traumatic Brain Injury Monitoring

The current body of literature supports further investigation into the role of NIRS in the guidance of precision medicine following TBI. In the pre-hospital setting, triaging guided by NIRS-based detection of hematomas may have the ability to improve personalized care plans to the point of impacting patient outcomes. Similarly, in the intraoperative setting, the role of NIRS to guide anesthetic management during surgery must be clearly delineated. Eventually, this may result in neuroprotective treatment algorithms guided by NIRS metrics measured in real-time. Progress in these areas will likely require large, multicentre, randomized, interventional trials.

In the neurocritical care setting, monitoring of cerebral oxygenation and CA by NIRS devices will need to be accurate and also be accompanied by proof that this data can inform better treatment strategies that are tailored to the individual patient. An example of this can be seen in the monitoring of cerebrovascular reactivity by ICP-based indices, where PRx is now being examined for its ability to identify personalized CPP targets (Kramer et al., 2019). Extensive studies to examine the implications of NIRS based indices of cerebrovascular reactivity on outcomes following TBI are already underway (Gomez et al., 2020a), while previous studies have shown agreement between CPP targets identified by PRx and those identified by NIRS-based indices (Zweifel et al., 2010). Eventually, we may see the neurocritical care management of TBI patients guided by multiple physiologic parameters measured with NIRS.

Beyond the acute phase, technologies such as fNIRS may help us understand how the brain recovers following TBI. With this information, progress in recovery maybe more objectively be tracked. Future rehabilitation strategies may even be individualized to patients based on their cerebral physiology as interrogated by NIRS.

In general, high-resolution physiome biomarkers in TBI show promise to improve diagnostic accuracy and predict the course of injury progression in real-time. However, the overwhelming volume of data presented to treating clinicians can be challenging to interpret. As such, progress has been made in identifying secondary injury patterns and categorizing different physiome biomarkers with machine learning algorithms that can analyze large complex biomedical datasets (Zeiler et al., 2017; Zeiler et al., 2018a; Zeiler et al., 2018b; Lee et al., 2019; Martinez and Stabenfeldt, 2019; van de Wijgert et al., 2021). As an exemplar, it has been shown that predictive machine learning algorithms can discover pediatric TBI biomarkers that are predictive of high-risk phenotypes in a moderate to severe injury cohort (Chong et al., 2015). Furthermore, hemodynamics have been explored as a possible critical biomarker of TBI, and it has been found with predictive classification algorithms that temporal and spatio-temporal features from the prefrontal cortex are candidate diagnostic markers of injury severity (Karamzadeh et al., 2016). While machine learning algorithms show potential in these previously explored smaller datasets, there is a need to consider large datasets to validate the biomarkers selected from these studies before they can be considered in clinical applications.

Finally, improvements in NIRS technology can minimize some of the previously noted limitations of this modality for continuous assessment of cerebral physiology in TBI patients. One such limitation of NIRS technology is its spatial resolution which is restricted by the number of detector optodes and how light scatters from the emitter to the detector optode. Multi-channel NIRS tackles this problem by increasing the optode density *via* an optode cap that places the optodes around a subject’s scalp (Chen et al., 2020). Results have shown that a multi-distance probe configuration gives better spatial resolution than a single distance probe configuration (Song et al., 2020). For the construction of a 3D fNIRS brain image, it has been shown that optodes combined in a bundled configuration increased the spatial resolution by describing brain activity more precisely than the conventional approach, which uses the Modified Beer-Lambert Law with a constant DPF. The bundled configuration contained 16 emitters and 16 detectors where each emitter can be combined with all the detectors, and the side-by-side configuration enabled the high precision detection of the active locations by the overlapping of the channels’ banana-shaped light paths (Nguyen and Hong, 2016). So, the spatial resolution is improved as the density of the optodes increases. Such work, employing advanced multi-channel high-frequency fNIRS devices for the development of continuous cerebrovascular reactivity mapping of the brain, is an ongoing focus of our lab.

## Conclusion

NIRS technology has a promising role in all phases of care in TBI. It may be utilized to extract clinically relevant biomarkers from the patient and enable greater insight into the physiome following injury. Further clinical research is required before the role of NIRS is fully understood. However, its non-invasive nature and ease of use make it an ideal modality to guide precision medicine in TBI. Further advances in technology may also reduce the current limitations of NIRS and open new avenues for clinical applications as well as areas of research in TBI.

## References

[B1] Abdalmalak A. Milej D. Norton L. Debicki D. B. Gofton T. Diop M. (2017). Single-session Communication with a Locked-In Patient by Functional Near-Infrared Spectroscopy. Neurophotonics 4, 040501. 10.1117/1.NPh.4.4.040501 29296627 PMC5741990

[B2] Al-Rawi P. G. Smielewski P. KirkpatrickPeter P. J. (2001). Evaluation of a Near-Infrared Spectrometer (NIRO 300) for the Detection of Intracranial Oxygenation Changes in the Adult Head. Stroke 32, 2492–2500. 10.1161/hs1101.098356 11692006

[B3] Arridge S. R. Cope M. Delpy D. T. (1992). The Theoretical Basis for the Determination of Optical Pathlengths in Tissue: Temporal and Frequency Analysis. Phys. Med. Biol. 37, 1531–1560. 10.1088/0031-9155/37/7/005 1631197

[B4] Bakker A. Smith B. Ainslie P. Smith K. (2012). Near-Infrared Spectroscopy. Applied Aspects of Ultrasonography in Humans. Editors Ainslie P. (InTech). Available at: http://www.intechopen.com/books/applied-aspects-of-ultrasonography-in-humans/near-infrared-spectroscopy

[B5] Bale G. Elwell C. E. Tachtsidis I. (2016). From Jöbsis to the Present Day: a Review of Clinical Near-Infrared Spectroscopy Measurements of Cerebral Cytochrome-C-Oxidase. J. Biomed. Opt. 21, 091307. 10.1117/1.JBO.21.9.091307 27170072

[B6] Bernard F. Gallagher C. Griesdale D. Kramer A. Sekhon M. Zeiler F. A. (2020). The CAnadian High-Resolution Traumatic Brain Injury (CAHR-TBI) Research Collaborative. Can. J. Neurol. Sci. 47, 551–556. 10.1017/cjn.2020.54 32174295

[B7] Bhambhani Y. Maikala R. Farag M. Rowland G. (2006). Reliability of Near-Infrared Spectroscopy Measures of Cerebral Oxygenation and Blood Volume during Handgrip Exercise in Nondisabled and Traumatic Brain-Injured Subjects. J. Rehabil. Res. Dev. 43, 845–856. 10.1682/jrrd.2005.09.0151 17436171

[B8] Büchner K. Meixensberger J. Dings J. Roosen K. (2000). Near-infrared Spectroscopy--Not Useful to Monitor Cerebral Oxygenation after Severe Brain Injury. Zentralbl Neurochir 61, 69–73. 10.1055/s-2000-8262 10986754

[B9] Buckley E. M. Parthasarathy A. B. Grant P. E. Yodh A. G. Franceschini M. A. (2014). Diffuse Correlation Spectroscopy for Measurement of Cerebral Blood Flow: Future Prospects. Neurophotonics 1, 011009. 10.1117/1.NPh.1.1.011009 25593978 PMC4292799

[B10] Budohoski K. P. Zweifel C. Kasprowicz M. Sorrentino E. Diedler J. Brady K. M. (2012). What Comes First? the Dynamics of Cerebral Oxygenation and Blood Flow in Response to Changes in Arterial Pressure and Intracranial Pressure after Head Injury. Br. J. Anaesth. 108, 89–99. 10.1093/bja/aer324 22037222 PMC3236021

[B11] Carney N. Totten A. M. O'Reilly C. Ullman J. S. Hawryluk G. W. Bell M. J. (2017). Guidelines for the Management of Severe Traumatic Brain Injury, Fourth Edition. Neurosurgery 80, 6–15. 10.1227/NEU.0000000000001432 27654000

[B12] Carp S. A. Farzam P. Redes N. Hueber D. M. Franceschini M. A. (2017). Combined Multi-Distance Frequency Domain and Diffuse Correlation Spectroscopy System with Simultaneous Data Acquisition and Real-Time Analysis. Biomed. Opt. Express 8, 3993–4006. 10.1364/BOE.8.003993 29026684 PMC5611918

[B13] Casati A. Fanelli G. Pietropaoli P. Proietti R. Tufano R. Danelli G. (2005). Continuous Monitoring of Cerebral Oxygen Saturation in Elderly Patients Undergoing Major Abdominal Surgery Minimizes Brain Exposure to Potential Hypoxia. Anesth. Analg 101, 740–contents. 10.1213/01.ane.0000166974.96219.cd 16115985

[B14] Chance B. Leigh J. S. Miyake H. Smith D. S. Nioka S. Greenfeld R. (1988). Comparison of Time-Resolved and -unresolved Measurements of Deoxyhemoglobin in Brain. Proc. Natl. Acad. Sci. U S A. 85, 4971–4975. 10.1073/pnas.85.14.4971 3393526 PMC281669

[B15] Chen W. L. Wagner J. Heugel N. Sugar J. Lee Y. W. Conant L. (2020). Functional Near-Infrared Spectroscopy and its Clinical Application in the Field of Neuroscience: Advances and Future Directions. Front. Neurosci. 14, 724. 10.3389/fnins.2020.00724 32742257 PMC7364176

[B16] Chong S. L. Liu N. Barbier S. Ong M. E. (2015). Predictive Modeling in Pediatric Traumatic Brain Injury Using Machine Learning. BMC Med. Res. Methodol. 15, 22. 10.1186/s12874-015-0015-0 25886156 PMC4374377

[B17] Cope M. (1991). The Development of a Near Infrared Spectroscopy System and its Application for Non Invasive Monitoring of Cerebral Blood and Tissue Oxygenation in the Newborn Infants. Doctoral thesis. London (UK): University of London. Available at: https://discovery.ucl.ac.uk/id/eprint/1317956/ (Accessed March 8, 2021).

[B18] Curtin A. Izzetoglu K. Reynolds J. Menon R. Izzetoglu M. Osbakken M. (2014). Functional Near-Infrared Spectroscopy for the Measurement of Propofol Effects in Conscious Sedation during Outpatient Elective Colonoscopy. Neuroimage 85 (1), 626–636. 10.1016/j.neuroimage.2013.07.009 23850462

[B19] Czosnyka M. Smielewski P. Kirkpatrick P. Laing R. J. Menon D. Pickard J. D. (1997). Continuous Assessment of the Cerebral Vasomotor Reactivity in Head Injury. Neurosurgery 41, 11–19. 10.1097/00006123-199707000-00005 9218290

[B20] Davies D. J. Clancy M. Dehghani H. Lucas S. J. E. Forcione M. Yakoub K. M. (2019). Cerebral Oxygenation in Traumatic Brain Injury: Can a Non-invasive Frequency Domain Near-Infrared Spectroscopy Device Detect Changes in Brain Tissue Oxygen Tension as Well as the Established Invasive Monitor? J. Neurotrauma 36, 1175–1183. 10.1089/neu.2018.5667 29877139

[B21] Davies D. J. Su Z. Clancy M. T. Lucas S. J. Dehghani H. Logan A. (2015). Near-Infrared Spectroscopy in the Monitoring of Adult Traumatic Brain Injury: A Review. J. Neurotrauma 32, 933–941. 10.1089/neu.2014.3748 25603012 PMC4492772

[B22] De Georgia M. A. (2015). Brain Tissue Oxygen Monitoring in Neurocritical Care. J. Intensive Care Med. 30, 473–483. 10.1177/0885066614529254 24710714

[B23] Delpy D. T. Cope M. van derZee P. Arridge S. Wray S. Wyatt J. (1988). Estimation of Optical Pathlength through Tissue from Direct Time of Flight Measurement. Phys. Med. Biol. 33, 1433–1442. 10.1088/0031-9155/33/12/008 3237772

[B24] Donnelly J. Czosnyka M. Adams H. Cardim D. Kolias A. G. Zeiler F. A. (2019). Twenty-Five Years of Intracranial Pressure Monitoring after Severe Traumatic Brain Injury: A Retrospective, Single-Center Analysis. Neurosurgery 85, E75–E82. 10.1093/neuros/nyy468 30476233

[B25] Dunham C. M. Ransom K. J. Flowers L. L. Siegal J. D. Kohli C. M. (2004). Cerebral Hypoxia in Severely Brain-Injured Patients Is Associated with Admission Glasgow Coma Scale Score, Computed Tomographic Severity, Cerebral Perfusion Pressure, and Survival. J. Trauma 56, 482–491. 10.1097/01.ta.0000114537.52540.95 15128117

[B26] Dunham C. M. Sosnowski C. Porter J. M. Siegal J. Kohli C. (2002). Correlation of Noninvasive Cerebral Oximetry with Cerebral Perfusion in the Severe Head Injured Patient: a Pilot Study. J. Trauma 52, 40–46. 10.1097/00005373-200201000-00009 11791050

[B27] Durduran T. Yodh A. G. (2014). Diffuse Correlation Spectroscopy for Non-invasive, Micro-vascular Cerebral Blood Flow Measurement. NeuroImage 85 (1), 51–63. 10.1016/j.neuroimage.2013.06.017 23770408 PMC3991554

[B28] Eggebrecht A. T. Ferradal S. L. Robichaux-Viehoever A. Hassanpour M. S. Dehghani H. Snyder A. Z. (2014). Mapping Distributed Brain Function and Networks with Diffuse Optical Tomography. Nat. Photon. 8, 448–454. 10.1038/nphoton.2014.107 PMC4114252 25083161

[B29] Esnault P. Boret H. Montcriol A. Carre E. Prunet B. Bordes J. (2015). Assessment of Cerebral Oxygenation in Neurocritical Care Patients: Comparison of a New Four Wavelengths Forehead Regional Saturation in Oxygen Sensor (EQUANOX®) with Brain Tissue Oxygenation. A Prospective Observational Study. Minerva Anestesiol 81, 876–884. 25415352

[B30] Froese L. Dian J. Batson C. Gomez A. Alarifi N. Unger B. (2020a). The Impact of Vasopressor and Sedative Agents on Cerebrovascular Reactivity and Compensatory Reserve in Traumatic Brain Injury: An Exploratory Analysis. Neurotrauma Rep. 1, 157–168. 10.1089/neur.2020.0028 33274344 PMC7703494

[B31] Froese L. Dian J. Batson C. Gomez A. Unger B. Zeiler F. A. (2020b). The Impact of Hypertonic Saline on Cerebrovascular Reactivity and Compensatory Reserve in Traumatic Brain Injury: An Exploratory Analysis. Acta Neurochir 162, 2683–2693. 10.1007/s00701-020-04579-0 32959342 PMC7505542

[B32] Froese L. Dian J. Gomez A. Zeiler F. A. (2021). Sedation and Cerebrovascular Reactivity in Traumatic Brain Injury: Another Potential Avenue for Personalized Approaches in Neurocritical Care? Acta Neurochir 163, 1383–1389. 10.1007/s00701-020-04662-6 33404872

[B33] Ghalenoui H. Saidi H. Azar M. Yahyavi S. T. Borghei Razavi H. Khalatbari M. (2008). Near-infrared Laser Spectroscopy as a Screening Tool for Detecting Hematoma in Patients with Head Trauma. Prehosp. Disaster Med. 23, 558–561. 10.1017/s1049023x00006415 19557974

[B34] Ghosh A. Highton D. Kolyva C. Tachtsidis I. Elwell C. E. Smith M. (2017). Hyperoxia Results in Increased Aerobic Metabolism Following Acute Brain Injury. J. Cereb. Blood Flow Metab. 37, 2910–2920. 10.1177/0271678X16679171 27837190 PMC5536254

[B35] Ghosh A. Tachtsidis I. Kolyva C. Highton D. Elwell C. Smith M. (2013). Normobaric Hyperoxia Does Not Change Optical Scattering or Pathlength but Does Increase Oxidised Cytochrome C Oxidase Concentration in Patients with Brain Injury. Adv. Exp. Med. Biol. 765, 67–72. 10.1007/978-1-4614-4989-8_10 22879016 PMC4038009

[B36] Gomez A. Dian J. Froese L. Zeiler F. A. (2020a). Near-Infrared Cerebrovascular Reactivity for Monitoring Cerebral Autoregulation and Predicting Outcomes in Moderate to Severe Traumatic Brain Injury: Proposal for a Pilot Observational Study. JMIR Res. Protoc. 9, e18740. 10.2196/18740 32415822 PMC7450363

[B37] Gomez A. Dian J. Zeiler F. A. (2020b). Continuous and Entirely Non-invasive Method for Cerebrovascular Reactivity Assessment: Technique and Implications. J. Clin. Monit. Comput. 35, 307–315. 10.1007/s10877-020-00472-4 31989415 PMC7382981

[B38] Gopinath S. P. Robertson C. S. Contant C. F. Narayan R. K. Grossman R. G. Chance B. (1995). Early Detection of Delayed Traumatic Intracranial Hematomas Using Near-Infrared Spectroscopy. J. Neurosurg. 83, 438–444. 10.3171/jns.1995.83.3.0438 7666220

[B39] Gopinath S. P. Robertson C. S. Grossman R. G. Chance B. (1993). Near-infrared Spectroscopic Localization of Intracranial Hematomas. J. Neurosurg. 79, 43–47. 10.3171/jns.1993.79.1.0043 8315468

[B40] Hashimoto K. Uruma G. Abo M. (2008). Activation of the Prefrontal Cortex during the Wisconsin Card Sorting Test (Keio Version) as Measured by Two-Channel Near-Infrared Spectroscopy in Patients with Traumatic Brain Injury. Eur. Neurol. 59, 24–30. 10.1159/000109257 17917454

[B41] Hernandez-Meza G. Izzetoglu M. Sacan A. Green M. Izzetoglu K. (2017). Investigation of Data-Driven Optical Neuromonitoring Approach during General Anesthesia with Sevoflurane. Neurophotonics 4, 041408. 10.1117/1.NPh.4.4.041408 28840160 PMC5562948

[B42] Herschel W. (1800a). Experiments on the Refrangibility of the Invisible Rays of the Sun. Philos. Trans. R. Soc. Lond. 90, 284–292.

[B43] Herschel W. (1800b). Investigation of the Powers of the Prismatic Colours to Heat and Illuminate Objects; with Remarks, that Prove the Different Refrangibility of Radiant Heat. To Which Is Added, an Inquiry into the Method of Viewing the Sun Advantageously, with Telescopes of Large Apertures and High Magnifying Powers. Philos. Trans. R. Soc. Lond. 90, 255–283.

[B44] Hessel T. W. Hyttel‐Sorensen S. Greisen G. (2014). Cerebral Oxygenation after Birth - a Comparison of INVOS and FORE ‐ SIGHT Near‐infrared Spectroscopy Oximeters. Acta Paediatr. 103, 488–493. 10.1111/apa.12567 24456266 PMC4112844

[B45] Hibino S. Mase M. Shirataki T. Nagano Y. Fukagawa K. Abe A. (2013). Oxyhemoglobin Changes during Cognitive Rehabilitation after Traumatic Brain Injury Using Near Infrared Spectroscopy. Neurol. Med. Chir (Tokyo) 53, 299–303. 10.2176/nmc.53.299 23708220

[B46] Highton D. Ghosh A. Tachtsidis I. Panovska-Griffiths J. Elwell C. E. Smith M. (2015). Monitoring Cerebral Autoregulation after Brain Injury: Multimodal Assessment of Cerebral Slow-Wave Oscillations Using Near-Infrared Spectroscopy. Anesth. Analg 121, 198–205. 10.1213/ANE.0000000000000790 25993387 PMC4957667

[B47] Holzschuh M. Woertgen C. Metz C. Brawanski A. (1997). Dynamic Changes of Cerebral Oxygenation Measured by Brain Tissue Oxygen Pressure and Near Infrared Spectroscopy. Neurol. Res. 19, 246–248. 10.1080/01616412.1997.11740807 9192374

[B48] Jöbsis F. F. (1977). Noninvasive, Infrared Monitoring of Cerebral and Myocardial Oxygen Sufficiency and Circulatory Parameters. Science 198, 1264–1267. 10.1126/science.929199 929199

[B49] Kahraman S. Kayali H. Atabey C. Acar F. Gocmen S. (2006). The Accuracy of Near-Infrared Spectroscopy in Detection of Subdural and Epidural Hematomas. J. Trauma 61, 1480–1483. 10.1097/01.ta.0000197616.10279.48 17159695

[B50] Karamzadeh N. Amyot F. Kenney K. Anderson A. Chowdhry F. Dashtestani H. (2016). A Machine Learning Approach to Identify Functional Biomarkers in Human Prefrontal Cortex for Individuals with Traumatic Brain Injury Using Functional Near-Infrared Spectroscopy. Brain Behav. 6, e00541. 10.1002/brb3.541 27843695 PMC5102640

[B51] Keller E. Nadler A. Niederer P. Yonekawa Y. Imhof H. G. (2003). A New Subdural Probe for Combined Intracranial Pressure (ICP) and Cerebral Blood Flow (CBF) Monitoring. Acta Neurochir (Wien) 145, 1111–1115. 10.1007/s00701-003-0102-6 14663569

[B52] Kessel B. Jeroukhimov I. Ashkenazi I. Khashan T. Oren M. Haspel J. (2007). Early Detection of Life-Threatening Intracranial Haemorrhage Using a Portable Near-Infrared Spectroscopy Device. Injury 38, 1065–1068. 10.1016/j.injury.2007.05.009 17716603

[B53] Kim M. N. Durduran T. Frangos S. Edlow B. L. Buckley E. M. Moss H. E. (2010). Noninvasive Measurement of Cerebral Blood Flow and Blood Oxygenation Using Near-Infrared and Diffuse Correlation Spectroscopies in Critically Brain-Injured Adults. Neurocrit. Care 12, 173–180. 10.1007/s12028-009-9305-x 19908166 PMC2844468

[B54] Kirkpatrick P. J. Smielewski P. Czosnyka M. Menon D. K. Pickard J. D. (1995). Near-infrared Spectroscopy Use in Patients with Head Injury. J. Neurosurg. 83, 963–970. 10.3171/jns.1995.83.6.0963 7490639

[B55] Kontojannis V. Hostettler I. Brogan R. J. Raza M. Harper-Payne A. Kareem H. (2019). Detection of Intracranial Hematomas in the Emergency Department Using Near Infrared Spectroscopy. Brain Inj. 33, 875–883. 10.1080/02699052.2019.1610796 31284787

[B56] Kontos A. P. Huppert T. J. Beluk N. H. Elbin R. J. Henry L. C. French J. (2014). Brain Activation during Neurocognitive Testing Using Functional Near-Infrared Spectroscopy in Patients Following Concussion Compared to Healthy Controls. Brain Imaging Behav. 8, 621–634. 10.1007/s11682-014-9289-9 24477579 PMC5080617

[B57] Kramer A. H. Couillard P. L. Zygun D. A. Aries M. J. Gallagher C. N. (2019). Continuous Assessment of “Optimal” Cerebral Perfusion Pressure in Traumatic Brain Injury: A Cohort Study of Feasibility, Reliability, and Relation to Outcome. Neurocrit. Care 30, 51–61. 10.1007/s12028-018-0570-4 29987688

[B58] Lakowicz J. R. Berndt K. (1990). Frequency-domain Measurements of Photon Migration in Tissues. Chem. Phys. Lett. 166, 246–252. 10.1016/0009-2614(90)80024-8

[B59] Lange F. Tachtsidis I. (2019). Clinical Brain Monitoring with Time Domain NIRS: A Review and Future Perspectives. Appl. Sci. 9, 1612. 10.3390/app9081612

[B60] Lassen N. A. (1959). Cerebral Blood Flow and Oxygen Consumption in Man. Physiol. Rev. 39, 183–238. 10.1152/physrev.1959.39.2.183 13645234

[B61] Le Roux P. Menon D. K. Citerio G. Vespa P. Bader M. K. Brophy G. M. (2014). Consensus Summary Statement of the International Multidisciplinary Consensus Conference on Multimodality Monitoring in Neurocritical Care: a Statement for Healthcare Professionals from the Neurocritical Care Society and the European Society of Intensive Care Medicine. Neurocrit. Care 21 (2), S1–S26. 10.1007/s12028-014-0041-5 25208678 PMC10596301

[B62] Leal-Noval S. R. Cayuela A. Arellano-Orden V. Marín-Caballos A. Padilla V. Ferrándiz-Millón C. (2010). Invasive and Noninvasive Assessment of Cerebral Oxygenation in Patients with Severe Traumatic Brain Injury. Intensive Care Med. 36, 1309–1317. 10.1007/s00134-010-1920-7 20502869

[B63] Lee J. K. Kibler K. K. Benni P. B. Easley R. B. Czosnyka M. Smielewski P. (2009). Cerebrovascular Reactivity Measured by Near-Infrared Spectroscopy. Stroke 40, 1820–1826. 10.1161/STROKEAHA.108.536094 19286593

[B64] Lee S. B. Kim H. Kim Y. T. Zeiler F. A. Smielewski P. Czosnyka M. (2019). Artifact Removal from Neurophysiological Signals: Impact on Intracranial and Arterial Pressure Monitoring in Traumatic Brain Injury. J. Neurosurg. 132, 1–9. 10.3171/2019.2.JNS182260 31075774

[B65] Leon-Carrion J. Dominguez-Roldan J. M. Leon-Dominguez U. Murillo-Cabezas F. (2010). The Infrascanner, a Handheld Device for Screening *In Situ* for the Presence of Brain Haematomas. Brain Inj. 24, 1193–1201. 10.3109/02699052.2010.506636 20715889

[B66] Maas A. I. Hukkelhoven C. W. Marshall L. F. Steyerberg E. W. (2005). Prediction of Outcome in Traumatic Brain Injury with Computed Tomographic Characteristics: a Comparison between the Computed Tomographic Classification and Combinations of Computed Tomographic Predictors. Neurosurgery 57, 1173–1182. 10.1227/01.neu.0000186013.63046.6b 16331165

[B67] Maas A. I. Stocchetti N. Bullock R. (2008). Moderate and Severe Traumatic Brain Injury in Adults. Lancet Neurol. 7, 728–741. 10.1016/S1474-4422(08)70164-9 18635021

[B68] Macmillan C. S. Andrews P. J. (2000). Cerebrovenous Oxygen Saturation Monitoring: Practical Considerations and Clinical Relevance. Intensive Care Med. 26, 1028–1036. 10.1007/s001340051315 11030158

[B69] Maillard J. Sologashvili T. Diaper J. Licker M. J. Keli Barcelos G. (2019). A Case of Persistence of Normal Tissue Oxygenation Monitored by Near-Infrared Spectroscopy (NIRS) Values Despite Prolonged Perioperative Cardiac Arrest. Am. J. Case Rep. 20, 21–25. 10.12659/AJCR.911399 30610182 PMC6330994

[B70] Marshall L. F. Marshall S. B. Klauber M. R. Clark M. v. B. Eisenberg H. M. Jane J. A. (1991). A New Classification of Head Injury Based on Computerized Tomography. J. Neurosurg. 75, S14–S20. 10.3171/sup.1991.75.1s.0s14

[B71] Martinez B. I. Stabenfeldt S. E. (2019). Current Trends in Biomarker Discovery and Analysis Tools for Traumatic Brain Injury. J. Biol. Eng. 13, 16. 10.1186/s13036-019-0145-8 30828380 PMC6381710

[B72] Matcher S. J. Kirkpatrick P. J. Nahid K. Cope M. Delpy D. T. (1995). “Absolute Quantification Methods in Tissue Near-Infrared Spectroscopy,” in Optical Tomography, Photon Migration, and Spectroscopy of Tissue and Model Media: Theory, Human Studies, and Instrumentation (Bellingham: International Society for Optics and Photonics), 486–495. 10.1117/12.209997

[B73] McLeod A. D. Igielman F. Elwell C. Cope M. Smith M. (2003). Measuring Cerebral Oxygenation during Normobaric Hyperoxia: a Comparison of Tissue Microprobes, Near-Infrared Spectroscopy, and Jugular Venous Oximetry in Head Injury. Anesth. Analg 97, 851–856. 10.1213/01.ane.0000072541.57132.ba 12933415

[B74] Milej D. He L. Abdalmalak A. Baker W. B. Anazodo U. C. Diop M. (2020). Quantification of Cerebral Blood Flow in Adults by Contrast-Enhanced Near-Infrared Spectroscopy: Validation against MRI. J. Cereb. Blood Flow Metab. 40, 1672–1684. 10.1177/0271678x19872564 31500522 PMC7370369

[B75] Nguyen H. D. Hong K. S. (2016). Bundled-optode Implementation for 3D Imaging in Functional Near-Infrared Spectroscopy. Biomed. Opt. Express 7, 3491–3507. 10.1364/BOE.7.003491 27699115 PMC5030027

[B76] Oda M. Yamashita Y. Nakano T. Suzuki A. Shimizu K. Hirano I. (1999). “Near-infrared Time-Resolved Spectroscopy System for Tissue Oxygenation Monitor,” in Optical Tomography and Spectroscopy of Tissue III (Bellingham: International Society for Optics and Photonics), 611–617. 10.1117/12.356809

[B77] Plachky J. Hofer S. Volkmann M. Martin E. Bardenheuer H. J. Weigand M. A. (2004). Regional Cerebral Oxygen Saturation Is a Sensitive Marker of Cerebral Hypoperfusion during Orthotopic Liver Transplantation. Anesth. Analg 99, 344. 10.1213/01.ANE.0000124032.31843.61 15271702

[B78] Rajaram A. Milej D. Suwalski M. Yip L. C. M. Guo L. R. Chu M. W. A. (2020). Optical Monitoring of Cerebral Perfusion and Metabolism in Adults during Cardiac Surgery with Cardiopulmonary Bypass. Biomed. Opt. Express 11, 5967–5981. 10.1364/BOE.404101 33149999 PMC7587277

[B79] Robertson C. S. Gopinath S. Chance B. (1997). Use of Near Infrared Spectroscopy to Identify Traumatic Intracranial Hemotomas. J. Biomed. Opt. 2, 31–41. 10.1117/12.261680 23014820

[B80] Robertson C. S. Zager E. L. Narayan R. K. Handly N. Sharma A. Hanley D. F. (2010). Clinical Evaluation of a Portable Near-Infrared Device for Detection of Traumatic Intracranial Hematomas. J. Neurotrauma 27, 1597–1604. 10.1089/neu.2010.1340 20568959

[B81] Rodriguez Merzagora A. C. Izzetoglu M. Onaral B. Schultheis M. T. (2014). Verbal Working Memory Impairments Following Traumatic Brain Injury: an fNIRS Investigation. Brain Imaging Behav. 8, 446–459. 10.1007/s11682-013-9258-8 24085609

[B82] Roldán M. Abay T. Y. Kyriacou P. A. (2020). Non-Invasive Techniques for Multimodal Monitoring in Traumatic Brain Injury: Systematic Review and Meta-Analysis. J. Neurotrauma 37, 2445–2453. 10.1089/neu.2020.7266 32821023

[B83] Rosenthal G. Furmanov A. Itshayek E. Shoshan Y. Singh V. (2014). Assessment of a Noninvasive Cerebral Oxygenation Monitor in Patients with Severe Traumatic Brain Injury. J. Neurosurg. 120, 901–907. 10.3171/2013.12.JNS131089 24484228

[B84] Rothoerl R. D. Faltermeier R. Burger R. Woertgen C. Brawanski A. (2002). Dynamic Correlation between Tissue PO2 and Near Infrared Spectroscopy. Acta Neurochir Suppl. 81, 311–313. 10.1007/978-3-7091-6738-0_79 12168334

[B85] Scholkmann F. Kleiser S. Metz A. J. Zimmermann R. Mata Pavia J. Wolf U. (2014). A Review on Continuous Wave Functional Near-Infrared Spectroscopy and Imaging Instrumentation and Methodology. NeuroImage 85 (1), 6–27. 10.1016/j.neuroimage.2013.05.004 23684868

[B86] Shafer R. Brown A. Taylor C. (2011). Correlation between Cerebral Blood Flow and Oxygen Saturation in Patients with Subarachnoid Hemorrhage and Traumatic Brain Injury. J. Neurointerv Surg. 3, 395–398. 10.1136/jnis.2010.004184 21990444

[B87] Smielewski P. Czosnyka M. Zweifel C. Brady K. Hogue C. Steiner L. (2010). Multicentre Experience of Using ICM+ for Investigations of Cerebrovascular Dynamics with Near-Infrared Spectroscopy. Crit. Care 14, P348. 10.1186/cc8580

[B88] Song X. Chen X. Chen L. An X. Ming D. (2020). Performance Improvement for Detecting Brain Function Using fNIRS: A Multi-Distance Probe Configuration with PPL Method. Front. Hum. Neurosci. 14, 569508. 10.3389/fnhum.2020.569508 33240063 PMC7677412

[B89] Suzuki S. Takasaki S. Ozaki T. Kobayashi Y. (1999). “Tissue Oxygenation Monitor Using NIR Spatially Resolved Spectroscopy,” in Optical Tomography and Spectroscopy of Tissue III (Bellingham: International Society for Optics and Photonics), 582–592. 10.1117/12.356862

[B90] Teasdale G. Jennett B. (1974). Assessment of Coma and Impaired Consciousness. A Practical Scale. Lancet 2, 81–84. 10.1016/s0140-6736(74)91639-0 4136544

[B91] Ter Minassian A. Poirier N. Pierrot M. Menei P. Granry J. C. Ursino M. (1999). Correlation between Cerebral Oxygen Saturation Measured by Near-Infrared Spectroscopy and Jugular Oxygen Saturation in Patients with Severe Closed Head Injury. Anesthesiology 91, 985–990. 10.1097/00000542-199910000-00018 10519501

[B92] Thavasothy M. Broadhead M. Elwell C. Peters M. Smith M. (2002). A Comparison of Cerebral Oxygenation as Measured by the NIRO 300 and the INVOS 5100 Near-Infrared Spectrophotometers. Anaesthesia 57, 999–1006. 10.1046/j.1365-2044.2002.02826.x 12358958

[B93] Thelin E. P. Raj R. Bellander B. M. Nelson D. Piippo-Karjalainen A. Siironen J. (2020). Comparison of High versus Low Frequency Cerebral Physiology for Cerebrovascular Reactivity Assessment in Traumatic Brain Injury: a Multi-center Pilot Study. J. Clin. Monit. Comput. 34, 971–994. 10.1007/s10877-019-00392-y 31573056 PMC7447671

[B94] Tisdall M. M. Tachtsidis I. Leung T. S. Elwell C. E. Smith M. (2008). Increase in Cerebral Aerobic Metabolism by Normobaric Hyperoxia after Traumatic Brain Injury. J. Neurosurg. 109, 424–432. 10.3171/JNS/2008/109/9/0424 18759572

[B95] Torricelli A. Contini D. Pifferi A. Caffini M. Re R. Zucchelli L. (2014). Time Domain Functional NIRS Imaging for Human Brain Mapping. Neuroimage 85 (1), 28–50. 10.1016/j.neuroimage.2013.05.106 23747285

[B96] Trofimov A. O. Kalentiev G. Voennov O. Grigoryeva V. (2016). Comparison of Cerebral Oxygen Saturation and Cerebral Perfusion Computed Tomography in Cerebral Blood Flow in Patients with Brain Injury. Adv. Exp. Med. Biol. 876, 145–149. 10.1007/978-1-4939-3023-4_18 26782206

[B97] van de Wijgert I. H. Jansen J. F. A. Tas J. Zeiler F. A. Voorter P. H. M. van Hal V. H. J. (2021). Semi-automated Computed Tomography Volumetry as a Proxy for Intracranial Pressure in Patients with Severe Traumatic Brain Injury: Clinical Feasibility Study. Acta Neurochir Suppl. 131, 17–21. 10.1007/978-3-030-59436-7_4 33839810

[B98] Verdecchia K. Diop M. Lee T. Y. St Lawrence K. (2013). Quantifying the Cerebral Metabolic Rate of Oxygen by Combining Diffuse Correlation Spectroscopy and Time-Resolved Near-Infrared Spectroscopy. J. Biomed. Opt. 18, 27007. 10.1117/1.JBO.18.2.027007 23389684

[B99] Vilkė A. Bilskienė D. Šaferis V. Gedminas M. Bieliauskaitė D. Tamašauskas A. (2014). Predictive Value of Early Near-Infrared Spectroscopy Monitoring of Patients with Traumatic Brain Injury. Medicina (Kaunas) 50, 263–268. 10.1016/j.medici.2014.10.001 25488161

[B100] Weatherall A. Skowno J. Lansdown A. Lupton T. Garner A. (2012). Feasibility of Cerebral Near-Infrared Spectroscopy Monitoring in the Pre-hospital Environment. Acta Anaesthesiol Scand. 56, 172–177. 10.1111/j.1399-6576.2011.02591.x 22236344

[B101] Weigl W. Milej D. Gerega A. Toczylowska B. Kacprzak M. Sawosz P. (2014). Assessment of Cerebral Perfusion in post-traumatic Brain Injury Patients with the Use of ICG-Bolus Tracking Method. Neuroimage 85 (1), 555–565. 10.1016/j.neuroimage.2013.06.065 23831529

[B102] Weigl W. Milej D. Janusek D. Wojtkiewicz S. Sawosz P. Kacprzak M. (2016). Application of Optical Methods in the Monitoring of Traumatic Brain Injury: A Review. J. Cereb. Blood Flow Metab. 36, 1825–1843. 10.1177/0271678X16667953 27604312 PMC5094301

[B103] Wray S. Cope M. Delpy D. T. Wyatt J. S. Reynolds E. O. (1988). Characterization of the Near Infrared Absorption Spectra of Cytochrome Aa3 and Haemoglobin for the Non-invasive Monitoring of Cerebral Oxygenation. Biochim. Biophys. Acta 933, 184–192. 10.1016/0005-2728(88)90069-2 2831976

[B104] Yu Y. Zhang K. Zhang L. Zong H. Meng L. Han R. (2018). Cerebral Near-Infrared Spectroscopy (NIRS) for Perioperative Monitoring of Brain Oxygenation in Children and Adults. Cochrane Database Syst. Rev. 1, CD010947. 10.1002/14651858.CD010947.pub2 29341066 PMC6491319

[B105] Zavriyev A. I. Kaya K. Farzam P. Farzam P. Y. Sunwoo J. Jassar A. S. (2021). The Role of Diffuse Correlation Spectroscopy and Frequency-Domain Near-Infrared Spectroscopy in Monitoring Cerebral Hemodynamics during Hypothermic Circulatory Arrests. JTCVS Tech. 7, 161–177. 10.1016/j.xjtc.2021.01.023 34318236 PMC8311503

[B106] Zeiler F. A. Cardim D. Donnelly J. Menon D. K. Czosnyka M. Smielewski P. (2018a). Transcranial Doppler Systolic Flow Index and ICP-Derived Cerebrovascular Reactivity Indices in Traumatic Brain Injury. J. Neurotrauma 35, 314–322. 10.1089/neu.2017.5364 29050524

[B107] Zeiler F. A. Donnelly J. Cardim D. Menon D. K. Smielewski P. Czosnyka M. (2018b). ICP versus Laser Doppler Cerebrovascular Reactivity Indices to Assess Brain Autoregulatory Capacity. Neurocrit. Care 28, 194–202. 10.1007/s12028-017-0472-x 29043544 PMC5948245

[B108] Zeiler F. A. Donnelly J. Menon D. K. Smielewski P. Zweifel C. Brady K. (2017). Continuous Autoregulatory Indices Derived from Multi-Modal Monitoring: Each One Is Not like the Other. J. Neurotrauma 34, 3070–3080. 10.1089/neu.2017.5129 28571485

[B109] Zeiler F. A. Ercole A. Cabeleira M. Zoerle T. Stocchetti N. Menon D. K. (2019). Univariate Comparison of Performance of Different Cerebrovascular Reactivity Indices for Outcome Association in Adult TBI: a CENTER-TBI Study. Acta Neurochir (Wien) 161, 1217–1227. 10.1007/s00701-019-03844-1 30877472 PMC6525666

[B110] Zeiler F. A. Ercole A. Czosnyka M. Smielewski P. Hawryluk G. Hutchinson P. J. A. (2020). Continuous Cerebrovascular Reactivity Monitoring in Moderate/severe Traumatic Brain Injury: a Narrative Review of Advances in Neurocritical Care. Br. J. Anaesth. 124, 440–453. 10.1016/j.bja.2019.11.031 31983411

[B111] Zhang Q. Ma H. Nioka S. Chance B. (2000). Study of Near Infrared Technology for Intracranial Hematoma Detection. J. Biomed. Opt. 5, 206–213. 10.1117/1.429988 10938785

[B112] Zweifel C. Castellani G. Czosnyka M. Helmy A. Manktelow A. Carrera E. (2010). Noninvasive Monitoring of Cerebrovascular Reactivity with Near Infrared Spectroscopy in Head-Injured Patients. J. Neurotrauma 27, 1951–1958. 10.1089/neu.2010.1388 20812789

[B113] Zweifel C. Lavinio A. Steiner L. A. Radolovich D. Smielewski P. Timofeev I. (2008). Continuous Monitoring of Cerebrovascular Pressure Reactivity in Patients with Head Injury. Neurosurg. Focus 25, E2. 10.3171/FOC.2008.25.10.E2 18828700

