# Supplementary material for: Near Infrared Spectroscopy for High-Temporal Resolution Cerebral Physiome Characterization in TBI: A Narrative Review of Techniques, Applications, and Future Directions
Source: Front Pharmacol. 2021 Nov 5;12:719501. doi: 10.3389/fphar.2021.719501 (PMC8602694; doi:10.3389/fphar.2021.719501)
Supplement: Supplementary file 1 [file Table1.DOCX]

**Table 1:** Summary of characteristics of various near infrared spectroscopy (NIRS) modalities.

| **Technique** | **Method** | **Cerebral Physiologic Metrics** | **Example of Device** | **Limitations** |
| --- | --- | --- | --- | --- |

| Spatially-resolved | Multiple light detectors measure the attenuation of NIR light at different wavelengths is measured on the illuminated tissue. Short and long detector distances separate superficial from deep tissue.  Currently used for bedside monitoring of TBI patients. | HbO  HHb  TOI  THI  rSO_2_ | NIRO-200NX  INVOS | Scattering parameters cannot be calculated directly, and are assumed from empirical tables. Therefore, calculations are accurate for relative changes in concentrations of chromophores instead of the absolute concentrations. |
| --- | --- | --- | --- | --- |
| Frequency-resolved | Using a light source, measurement is made of the detected light intensity along with its phase shift and modulation depth with respect to the input light. This information is used to quantify the degree of scatter in the tissue. | HbO  HHb  cStO_2_ | FORE-SIGHT | If only one frequency is used, there will be less information provided about the tissue. |
| Time-resolved | A picosecond laser source is used to generate ultrashort pulses, light is input in tissues, and the intensity of the emergent light from the tissue is detected as the temporal point spread function (TPSF) with picosecond resolution.  It contains information about changes in light absorption at different depths. | HbO  HHb  HbT  TPSF  SO_2_ | TRS-10 | Using a streak camera gives the system limited dynamic range, is expensive and difficult to use in a clinical environment due to its large size.  The time-correlated photon counting system detector is limited by its low speed. |
| Diffuse correlation spectroscopy | Temporal fluctuations of the reflected NIR light are used to quantify the cerebral blood flow by detecting photon arrival of these fluctuations by intensity temporal autocorrelation function.  By combining NIRS with DCS, the cerebral tissue oxygen metabolic rate (CMRO_2_) can be quantified. | CBF  CMRO_2_ | MetaOx | DCS is sensitive to superficial cortical regions in adults so increasing the penetration depth with larger source-detector distance leads to low signal-to-noise ratio (SNR) and grouping multiple detector fibers is an expensive option .  The combination of low SNR and presence of hair and/or dark skin, can increase the acquisition time and reduce temporal resolution. |

*CMRO­_2_ = Cerebral tissue oxygen metabolic rate; cStO2 = Regional cerebral tissue oxygenation; DCS = Diffuse correlation spectroscopy; HbO = Oxygenated hemoglobin; HHb = Deoxygenated hemoglobin; NIRS = Near infrared spectroscopy; rSO_2 ­_= Regional cerebral oxygen saturation; SO_2_ = Oxygen saturation; TBI = Traumatic brain injury; THI = Total hemoglobin index; TPSF =* *Temporal point spread function; TOI = Tissue oxygen index.*
